# Supplementary material for: Identification of photoperiod-induced specific miRNAs in the adrenal glands of Sunite sheep (Ovis aries)
Source: Front Vet Sci. 2022 Jul 22;9:888207. doi: 10.3389/fvets.2022.888207 (PMC9354845; doi:10.3389/fvets.2022.888207)
Supplement: Supplementary file 1 [file Data_Sheet_1.ZIP › Supplementary file/miRDeep2 file.zip!/miRDeep2 file/Know_Structure/SPLP42A1/oar-mir-433.pdf]

miRBase precursor : oar-mir-433
Total read count : 1587
oar-miR-433-5p read count : 1
oar-miR-433-3p read count : 1586
remaining reads : 0

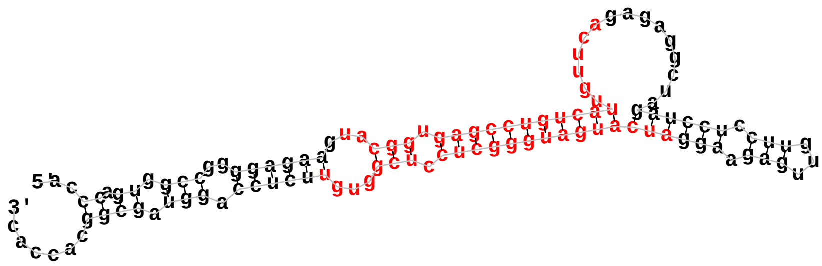

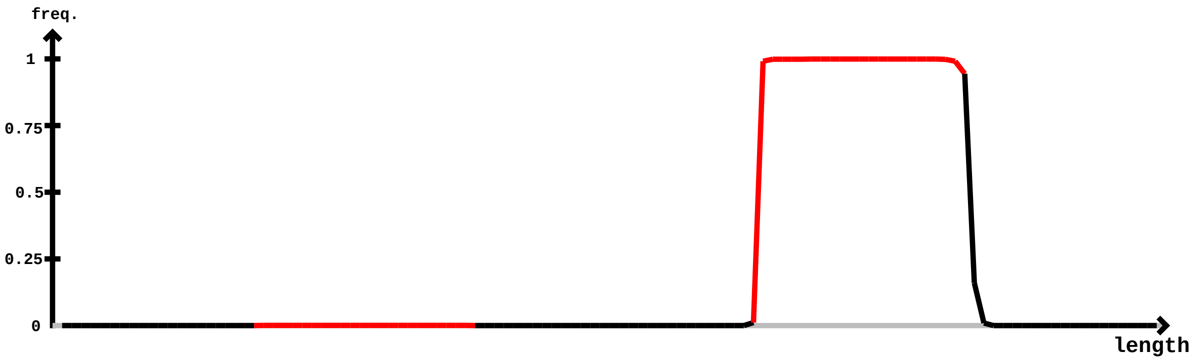


```
                                      oar-miR-433-5p
                                                                          oar-miR-433-3p
5'- acccaguggccggggagaaguacggugagccugucauuguucagagaggcuagauccuccuuguugagaagggaucaugaugggcuccucggugguucuccagguagcggcaccac   -3'    exp
    ..((.((.(((..((((((...(((.(((((((((((...............((((((.(((...))).))))))))))))))))).)))...)))))).))).))))......    reads    mm    sample
    ....................uacggugagccugucauuguuc........................................................................    1        0     seq
    .......................................................................gaucaugaugggcuccucggug.....................    1        0     seq
    .......................................................................gaucaugaugggcuccucggugu....................    15       0     seq
    .......................................................................gaucaugaugggcuccucgguguu...................    2        0     seq
    ........................................................................aucaugaugggcuccucgg.......................    2        0     seq
    ........................................................................aucaugaugggcuccucggu......................    11       0     seq
    ........................................................................aucaugaugggcuccucggug.....................    73       0     seq
    ........................................................................aucaugaugggcuccucggugu....................    1219     0     seq
    ........................................................................aucaugaugggcuccucgguguu...................    235      0     seq
    ........................................................................aucaugaugggcuccucgguguuc..................    15       0     seq
    .........................................................................ucaugaugggcuccucggugu....................    11       0     seq
    .........................................................................ucaugaugggcuccucgguguu...................    1        0     seq
    .............................................................................gaugggcuccucgguguu...................    1        0     seq
```
